# Development and validation of an emotion regulation training intervention for primary education students

**DOI:** 10.3389/fpsyg.2026.1764661

**Published:** 2026-03-16

**Authors:** Peter Vogl, Michael Methlagl, Barbara Hanfstingl

**Affiliations:** 1 Department of Sport and Human Movement Science, University of Vienna, Vienna, Austria; 2 Department of Primary Education, University College of Teacher Education Vienna, Vienna, Austria; 3 Training and Sports Sciences, University of Applied Sciences Wiener Neustadt, Wiener Neustadt, Austria; 4 Department of Psychology, University of Klagenfurt, Klagenfurt am Wörthersee, Austria

**Keywords:** education, emotion regulation, intervention, teacher, training

## Abstract

**Background:**

The present intervention study aims to implement a course concept (ERTL) for emotion regulation (ER) in higher-education teacher training in Austria. ERTL was designed to prepare participants for emotionally charged situations in their future profession.

**Objective:**

This study investigated the impact of an ER training program for primary pre-service teachers (ERTL). The development of ERTL takes into account the social environment encountered by pre-service teachers, focusing on training both intra- and interpersonal ER strategies (ERS). This is done within the context of emotional labor demands, socially challenging classroom situations, and individual experiences and preferences.

**Methods:**

Quantitative data were collected using a quasi-experimental design. The intervention (*n* = 86) and control (*n* = 42) groups completed questionnaires assessing intra- (RESS) and interpersonal (IERQ) ERS at two measurement points (pre- and post-intervention). Due to curricular guidelines, the intervention spanned 3 weeks across three modules.

**Results:**

The evaluation showed significantly increased arousal control (*p* = 0.03, robust ES = 0.74) and reappraisal (*p* = 0.011, ω^2^ = 0.012), as well as decreased rumination (*p* = 0.017, ω^2^ = 0.012), among students in the intervention group, with no significant changes in the control group. No significant effects were found in the interpersonal ER scales.

**Conclusion:**

Based on these findings, the CSI model (Context-Situation-Individual), derived from the ERTL design, not only provides a promising framework for the intervention but also serves as a bridge between theory and practice for the application of ERS by teachers in the classroom. The further development of the ERTL prototype into five modules with more exercises on interpersonal ERS will provide additional insights.

## 1 Introduction

Over the last decades, research has consistently highlighted the emotional nature of teaching (e.g., Frenzel et al., 2020; Hargreaves, 1998; Pekrun and Linnenbrink-Garcia, 2014; Sutton, 2004); however, there is a notable lack of training programs that assist pre-service teachers in managing emotions in challenging situations (Pekrun et al., 2018; Schelhorn and Kuhbandner, 2021; Taxer and Gross, 2018). Although schools provide stress management interventions (Lehr et al., 2013), mindfulness trainings (Hwang et al., 2019), and social and emotional learning programs (Carstensen et al., 2019; Hoffmann et al., 2020), and emotional intelligence trainings (Gilar-Corbi et al., 2018; Kotsou et al., 2018; Özdemir Cihan and Dilekmen, 2024), there remains a need for specific training for pre-service teachers in applying emotion regulation strategies (ERS) in teaching contexts (Taxer and Gross, 2018). Teachers’ emotion regulation is crucial for their well-being (Buric and Moe, 2020), enhances positive student-teacher relationships (Ocak Karabay, 2019), supports adaptive anger management (Deng et al., 2022), influences classroom effectiveness, and ultimately leads to improved student learning outcomes (Frenzel et al., 2021). However, effective emotion regulation (ER) by teachers depends on an adaptive set of ERS (Li et al., 2025), and actual use is shaped by contextual and situational factors (Haines et al., 2016; Lauermann, 2024; Pekrun and Marsh, 2022) as well as personal preferences and traits (Li and Akram, 2023). The development of the intervention presented in this study incorporates the ongoing discourse concerning the contextual, situational, and individual prerequisites for employing specific ERS (Harley et al., 2019; Frenzel et al., 2015). Additionally, the adaptable application of a comprehensive array of strategies across various situations, with consideration of the school context and its respective cultural dynamics, alongside the foundations of the emotional labor (EL) and emotion regulation (ER) traditions, with reference to the Austrian education system, and the prevailing understanding of the profession, informs the design of the current emotion regulation training program for pre-service teachers (ERTL – the acronym stands for the German initials of the program: **E**motions**R**egulations**T**raining für **L**ehrpersonen). Participants in the ERTL program develop their scope for ER in teaching through multidisciplinary, theory- and practice-oriented activities that support students in accepting emotions as an integral part of teaching and in developing a broad repertoire of ERS tailored to context, situation, and individual across three modules. Implementing interventions such as ERTL in teacher education programs can help aspiring teachers better cope with the emotional demands of teaching (Xie, 2021) and support their professional development (Li and Akram, 2023). Moreover, by equipping teachers with the tools to manage their negative emotions effectively, educational institutions can foster a healthier, more productive teaching environment (Tong, 2025).

## 2 Literature review

This literature review covers foundational theories and existing work on teachers’ emotion regulation in the context of developing a holistic training program. Previous interventions are often focused on a single aspect, technique, or target audience—helping teachers cope with stress (Schussler et al., 2018) and fostering resilience (Jennings et al., 2019); using mindfulness-based practices (Gouda et al., 2016; Tarrasch, 2019); or cultivating ER in students (Hoffmann et al., 2020) – and fail to address the importance of the social world (Boiger and Mesquita, 2012; Fischer and van Kleef, 2010).

Traditional educational research has often overlooked contextual and situational variables (Pekrun and Marsh, 2022), underscoring the need to integrate multiple theoretical perspectives to understand complex phenomena such as teachers’ ER (Frenzel et al., 2021). Indeed, Yin et al. (2025) identified three theoretical perspectives on teachers’ emotion regulation—individual, interaction, and context-focused – all of which is essential for designing and implementing an effective intervention in higher teacher education. In addition, Li and Akram (2023) found a significant relationship between ER and teachers’ professional development, suggesting that teacher education programs should incorporate ER knowledge into their curricula (see also Xu et al., 2026).

The individual perspective is grounded in the process model of emotion regulation (Gross, 1998; Gross, 2015). Emotion regulation, as defined by Gross (1998, p. 275), “refers to the processes by which individuals influence which emotions they have, when they have them, and how they experience and express these emotions”. This emotion regulation model outlines five families of intrapersonal ERS within the emotional process, distinguishing between antecedent-focused strategies—such as situation selection, situation modification, attentional deployment, and cognitive change—and response-focused strategies, specifically response modulation. Antecedent-focused ERS have been identified as effective in enhancing primary teachers’ emotional well-being (Li et al., 2023) and in reducing the expression of negative emotions (Jiang et al., 2016). In educational contexts, teachers are frequently exposed to high stress and emotional exhaustion, which can negatively affect their job satisfaction (Braun et al., 2020; Yin et al., 2016). Research has suggested that teachers with better emotion regulation abilities tend to demonstrate increased job satisfaction and a more positive outlook towards their profession (Brackett et al., 2010). Li et al. (2022) reports a direct relationship between teachers’ mindfulness, immunity, and work engagement, indicating that mindfulness enhances teachers’ ability to manage emotions and cope with contextual and situational adversities. Although teachers from different cultures share a similar understanding of work engagement (Wang et al., 2025), individual interpretations of contexts and situations shape experiences and the expression of emotions in teaching (Haines et al., 2016). This individual perspective is the first pillar of the ERTL program.

Given the rapidly changing situations and interactions teachers face daily in their professional lives, training programs that promote the flexible application of various ERS are essential (Aldao et al., 2014). Consequently, a more comprehensive approach that incorporates social factors within the context of relationship dynamics (Hagenauer and Raufelder, 2021) has been identified in interpersonal emotion regulation (IER) (Zaki and Williams, 2013). This concept describes the emotion regulation processes that regulate emotions through others and are therefore necessarily embedded in social situations. A diary pre-study (Vogl et al., 2025a) for the development of this intervention found four major situation categories relevant for Austrian pre-service primary teachers: behavioral issues (Kwok, 2021; McLean et al., 2019), instructional issues (Liu et al., 2023), emotional and social issues (Taylor and Smith, 2017) as well as cultural-, diversity and inclusion issues (Li et al., 2022). A limited range of situation-appropriate ERS (De France and Hollenstein, 2017) can lead to conflicts in daily life (Barnow et al., 2016). Since emotion regulation is not a one-size-fits-all solution, understanding the situation in which teachers individually employ different ERS is crucial (McRae and Gross, 2020). For instance, while in moderate stress situations, teachers may use cognitively focused strategies; in high-stress situations, others might rely more on behavioral modifications to manage classroom emotions (Burić et al., 2017; Li et al., 2023). Furthermore, the range of subjects primary teachers teach in Austria is very broad, and some pre-service teachers might favor one subject over the others, leading to individually different daily emotional experiences (Pekrun et al., 2023). This less-explored theoretical strand (Li et al., 2025) forms the second key concept of the ERTL intervention.

Context-focused theories are often connected to the emotional labor framework (Hochschild, 1979, 2012) and a sociological view on emotion. The former conceptualizes emotional labor as a process in which workers either genuinely experience (deep acting) or feign (surface acting) specific emotions to accomplish professional tasks (Kruml and Geddes, 2000). This phenomenon is characterized as a job requirement that alienates workers from authentic emotions (Grandey and Melloy, 2017). Although deep acting requires more effort, surface acting causes more harm to workers (Hülsheger and Schewe, 2011). The effort of an individual displaying organizationally appropriate emotion may lead to stress and burnout (Sutton, 2004). The expression of emotion depends on meeting culturally appropriate etiquette. The rules for controlling this display and what evokes or elicits an emotion differ between cultures and contexts (Ekman et al., 1969). Often termed “cultural display rules” (Matsumoto and Ekman, 2010, p. 342), these are rules acquired early in life, heavily shaped by social circumstances, and they “dictate the management and modification of emotional displays” (Matsumoto and Ekman, 2010, p. 343). Within the educational context, display rules inform the appropriate emotional expressions for both supportive and disciplinary emotion-regulation strategies (Barber et al., 2010). A significant consideration in the teaching profession is whether educators genuinely express, hide, fake, or mask their emotions (Taxer and Frenzel, 2015). A pivotal question in research on emotion regulation strategies is their effectiveness in matching outward emotional display with the pertinent display rules. In such instances, individuals are not required to alter their emotional experiences to regulate their expressive behavior (Barber et al., 2010). This internal struggle renders teaching an emotionally demanding profession (Hargreaves, 1998). Furthermore, teachers perceive display rules as shaped by the broader school context and culture; by specific locations within the school building; and by gender, identity, and teachers’ understandings of their professional role (Stark and Bettini, 2021). Alternatively, social norms have also been conceptualized in terms of how emotions, encompassing processes of production, embodiment, and interpretation of meanings, can only be understood within the particular social conventions on which they are based (Zembylas, 2007). Teachers’ job demands entail interactions with students, colleagues, and parents, as well as acting in accordance with the school’s organizational culture (Wharton, 2009). Consequently, in the context of teaching, display rules influence teachers’ emotion regulation.

In conclusion, it is important to note the conceptual imprecision of the terms used to describe teachers’ emotional management. The umbrella concept of ER (Aldrup et al., 2023) encompasses both overlaps and distinctions among traditions, approaches, and terms. The ability to choose an appropriate adaptive strategy from a broad set of ERS can be seen as part of emotional intelligence (Aldrup et al., 2023; Mayer et al., 2008), whereas teachers´ emotional competence (Savina et al., 2025) includes students’ emotional and social learning.

These theoretical perspectives inform the development of the intervention and the preparation of pre-service teachers to apply intra- and interpersonal ERS within the context of school requirements, helping them manage challenging classroom situations while considering individual needs and preferences, thereby bridging the gap between theory and practice.

## 3 Development of the ERTL intervention

The development of the ERTL intervention is guided by the hypothesis that there are no universal ERS that are always “good”; rather, contextual and situation-specific factors, as well as individual preferences and self-efficacy regarding the use of a specific ERS, are decisive for the effectiveness of teachers’ ER (Zhang et al., 2022). The ERTL training aims to help students understand and believe in their ability to influence and manage their negative emotions (Chang, 2020; Nalipay et al., 2021). The program is designed with two complementary goals, inspired by existing clinical programs (Sulz, 2011, p. 132) and adapted for educational settings:

- Replacing unrealistic interpretations of situations with realistic ones, thereby eliminating the basis for dysfunctional feelings in school contexts and challenging situations.
- Transforming feelings into context-appropriate action impulses that facilitate socially functional and professional interactions and relationships with students.

To achieve these goals, the intervention model is structured into three modules: context, situations, and individual (Vogl et al., 2025b) to develop pre-service teachers’ intra- and interpersonal ERS. Emotions are context-dependent and individually constructed (Barrett, 2017). Consequently, it is crucial to consider mental models concerning everyday school situations and one’s self-perception as a teacher in relation to emotional experiences (Ocak Karabay, 2019). The intervention modules contextualize ERS by addressing the organizational and emotional labor demands inherent in educational institutions, social dynamics at both the classroom and interaction levels, and personal reflections on the practical applicability of ERS. The training process is rooted in the challenging situations students face during their teaching practicum or professional careers. The ERTL setting facilitates peer exchanges guided by an experienced academic trainer with professional training in group pedagogy and psychology, tailored to emotionally demanding scenarios.

**Figure 1:**
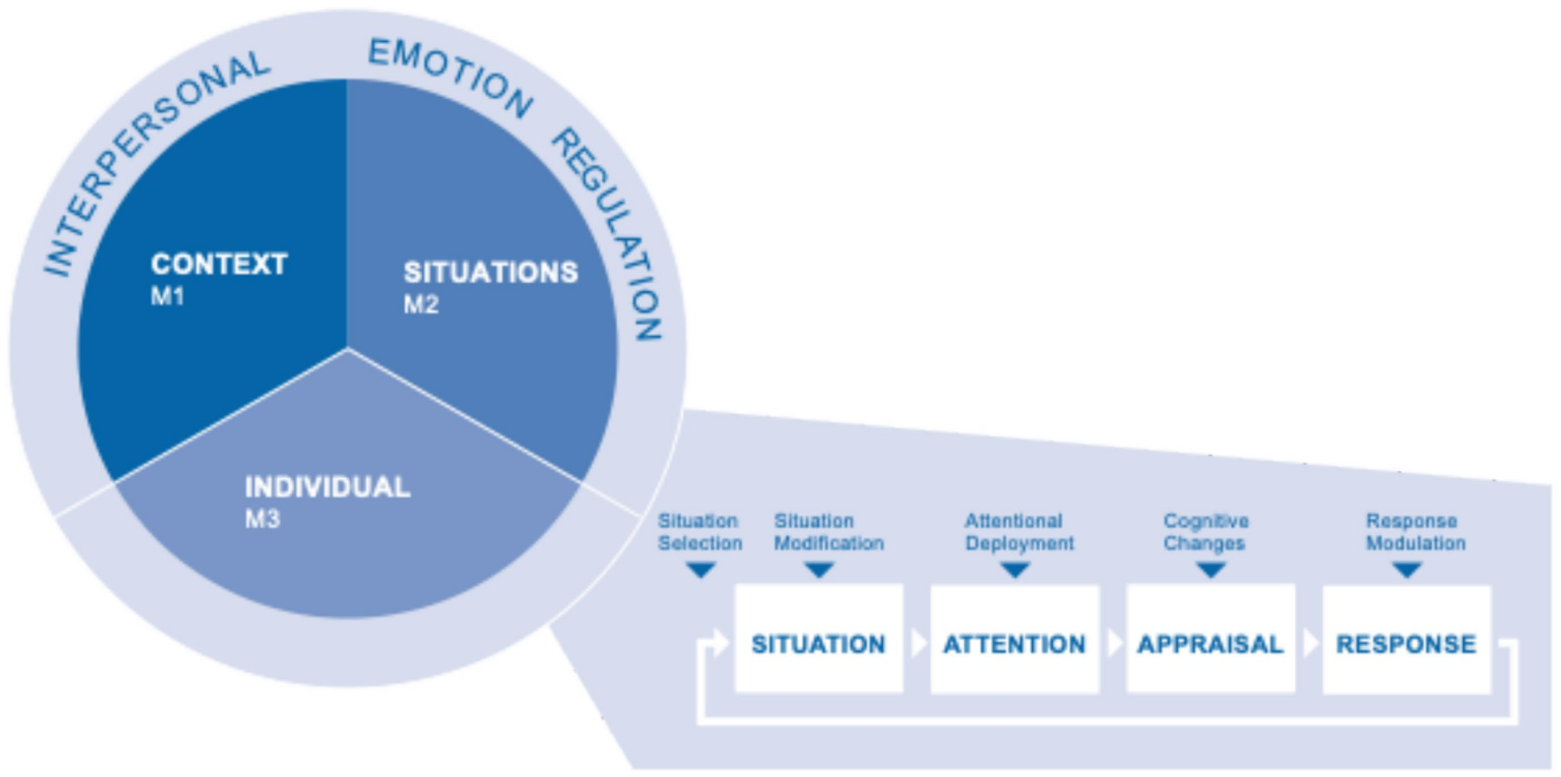
CSI (Context-Situation-Individual) model based on interpersonal ER (Zaki and Williams, 2013) and intrapersonal ER (Gross, 1998).

Within the context of one’s professional understanding, the group discusses emotional experiences to achieve relief. This aspect is shown in the intervention model (see Figure 1) by an outer framing ring (interpersonal ER) integrated into the training.

The sequence of the modules follows a top-down approach wherein the context of the profession represents the widest frame, followed by situations in which individuals regulate their emotions.

### 3.1 Module 1: context (M1)

The cultural dimensions of schools, including associated values and display rules (Hochschild, 2012), play a pivotal role in shaping the perception of emotions (Chang, 2020; Koopmann-Holm and Matsumoto, 2011). Adopting a realistic perspective on emotions facilitates re-evaluating personal experiences in the educational context, thereby fostering acceptance of one’s emotional states (Chang, 2009). In this module, students are encouraged to discuss the emotional labor demands inherent in their prospective careers. By reflecting on and destigmatizing emotions within an organizational framework, students can mitigate misinterpretations and effectively regulate emotions. By using the schema of antinomies in pedagogical action (Helsper, 2001), students can contextualize their emotional experiences. Moreover, the theoretical exploration of emotions, encompassing processes such as appraisal, action tendency, bodily reaction, expression, and feeling components (Gillioz et al., 2016; Scherer and Moors, 2019), provides the foundational basis for the introduction of Gross’s process model of emotion regulation and intrapersonal ERS.

### 3.2 Module 2: situations (M2)

Module 2 focuses on pre-service teachers’ personal experiences of challenging classroom situations. In a workshop format, participants provide their experiences (case bringers) and develop their individual intra- and interpersonal ERS to manage these emotional classroom situations. Students work on subject-specific behavioral issues, instructional issues, emotional and social issues, as well as cultural, diversity, and inclusion issues (Vogl et al., 2025a). Furthermore, participants discuss triggers of negative emotions and learn to use adaptive ERS (Deng et al., 2022). The teacher-student relationship and the associated professional understanding regarding proximity and distance issues are central to this module. The finding that the more teachers care for their students, the higher the risk of professional frustration (Chang, 2009) serves as a starting point for reflecting on the role and function of teachers. Lastly, the intrinsic response-independent mechanism of interpersonal ER (Zaki and Williams, 2013), in terms of labeling one’s feelings and integrating emotional vocabulary in the classroom (Ikeda, 2023), is trained in Module 2.

### 3.3 Module 3: individual (M3)

Module 3 concentrates on the individual-specific needs and preferences associated with emotion regulation strategies. Participants engaged in techniques for managing bodily arousal and practiced acceptance. Maladaptive strategies, such as avoidance, rumination, and suppression, are contrasted with adaptive strategies, such as cognitive change and problem-solving (situation modification) (Aldao and Schweizer, 2009). Rumination often arises from cognitive self-narratives that perpetually assess the future or past (Guendelman et al., 2017). Meditative practices that promote acceptance can mitigate such obstructive thoughts (Tang et al., 2015). Participants are encouraged to reflect on aspects they can control and to develop constructive hopelessness (Berking and Znoj, 2008) (i.e., ERS cognitive change) for contexts and situations beyond their influence. The framework of psychological flexibility emphasizes the ability to be present and open to difficult thoughts and feelings (Hayes, 2019). In this module, students participate in practical mental and physical exercises, which are then discussed in terms of their potential and limitations for teaching practice.

## 4 Aim of the study and research question

The present study aims to investigate the effects of a customized intervention program tailored for primary education students, with an emphasis on promoting adaptive emotion regulation strategies relevant to their future professional endeavors. The findings are anticipated to make a substantial contribution to the ongoing refinement of the intervention and to inform future training and professional development initiatives.

It was hypothesized that participants in the ERTL program would show (a) increased use of the ERS engagement, arousal control, distraction, and reappraisal, as well as (b) decreased use of ERS suppression and rumination in intrapersonal emotion regulation. Additionally, it was suggested that the intervention group would demonstrate (c) improved social modeling, perspective-taking, positive affect, and soothing. Furthermore, it was hypothesized that over three weeks, there would be significant changes compared to the control group. The following research question guided the study:

*RQ:* Does the intervention lead to significant changes in the participants’ intra- and interpersonal emotion regulation over time compared to the control group?

## 5 Materials and methods

### 5.1 Participants

The participants in this study were students enrolled in the primary education program at the Austrian University College of Teacher Education, Vienna. The ERTL program was embedded in a university course in which the participants self-enrolled. The students were able to choose the ERTL intervention from a pool of different course offerings in the eighth semester. The control group included students enrolled in other courses within the same study program at the Austrian University College of Teacher Education, Vienna.

The intervention study involved 126 students (16 male, 110 female, 2 non-binary; M_age_ = 24.45 years, SD_age_ = 4.01 years), who were assigned to an intervention group (*n* = 86; M_age_ = 25.12 years, SD_age_ = 4.07 years; 83% female) and a control group (*n* = 42; M_age_ = 23.1 years, SD_age_ = 3.55 years; 93% female). Baseline age differences between the intervention- and control group groups were found (U = 995.5; *p* < 0.001, *r_rb_* = 0.449; Mdn _IG_ = 24 years; Mdn_CG_ = 22 years). There were no significant gender differences between the intervention and control group (χ^2^(2) = 3.611; *p* = 0.164).

The age differences between the two groups can be explained by progress during one’s studies. The ERTL intervention was offered only to students in their final year; control group students were asked to complete the questionnaires as early as the third year to recruit enough participants.

Enrollment figures for the intervention group indicate strong student interest in the ERTL program. Recruiting participants for the control group was more difficult, as evidenced by their substantially lower voluntary questionnaire response rates.

### 5.2 Measures

#### 5.2.1 Regulation of emotion system survey

The RESS self-report questionnaire (De France and Hollenstein, 2017; De France and Hollenstein, 2019) was used to assess six intrapersonal ER strategies:

- Cognitive strategies: distraction (example item: “Immediately working on something to keep myself busy”), rumination (example item: “Thinking repeatedly about what was bothering me”), and reappraisal (example item: “Trying to see the emotional event from a different perspective”).
- Arousal strategy: arousal control (example item: “Trying to slow my heart rate and breathing”).
- Behavioral strategies: suppression (example item: “Acting like I was not upset”) and engagement (example item: “Showing my feelings”).

Participants are required to respond to the 38 items on a five-point scale (1 = never to 5 = always), completing the prompt “At the time I experience a negative emotion, I usually respond to it right away by …” (De France and Hollenstein, 2017, p. 205). Higher scores indicate that the ER strategy is used more often. One item was removed because its wording was identical to another item during translation into German.

#### 5.2.2 Interpersonal emotion regulation questionnaire

To assess emotion regulation through interactions with others (interpersonal ER), the IERQ (Hofmann et al., 2016) was used. It consists of 20 items (5-point Likert-scale; 1 = does not apply to me at all to 5 = applies very much to me) that represent the four IER strategies:

- Enhancing positive affect (example item: “I like being around others when I’m excited to share my joy”), perspective taking (example item: “Having people remind me that others are worse off helps me when I’m upset”),
- Soothing (example item: “I look for other people to offer me compassion when I’m upset”),
- Social modeling (example item: “It makes me feel better to learn how others dealt with their emotions”).

#### 5.2.3 Procedure and study design

After developing the training program, ERTL was incorporated into the regular Bachelor’s curriculum for primary school teaching during the eighth semester as a “practice-oriented elective course“. The ERTL was implemented from June 2023 to December 2024. The course was conducted in group sessions, with each cohort consisting of approximately 15 to 25 students and held weekly over three sessions. According to the institutional framework and guidelines, the course included three sessions, each lasting 135 min (M1–M3). These intervention group meetings (UE) were held weekly for three weeks, with each cohort comprising approximately 15 to 25 students. The control group was made up of students registered in other courses within the same degree program. Participants were randomly selected from various Bachelor of Primary Education courses conducted simultaneously with the intervention at the University College of Teacher Education Vienna and asked to join the control group and complete the survey at two measurement points (pre- and post-intervention).

The intervention protocol (see Table 1) outlines the learning outcomes, theoretical foundations, and activities of the three ERTL modules, including the time allocation for each component.

**Table 1:** Intervention protocol.

Intervention Group				Control group
Modules	Learning outcomes	Theory	Exercises and activities	
Module 1: Context total time 135 min	Students recognize emotions in the teaching profession as an interdisciplinary phenomenon, regulating them in a context-specific and situation-specific manner.	1. Emotion Components 2. ER 2.1 & EL Traditions in Teaching 3. Organizational demands, display rules and teacher role 4. Antinomies in pedagogical action Duration: Approximately 60 min	(a) Guide discussion (dyad, triad, group) of students’ experiences of 1.-4. during their practicum and how the respective theories impact one’s own emotions; (b) Watching a scene from a Netflix series (Ben’s school teacher) to reflect on 1.-4.: writing down dynamics & insights, discussing it with peers in a small group, then plenum. Duration: Approximately 75 min	Participants were randomly selected from various Bachelor of Primary Education courses conducted simultaneously with the intervention at the university college of teacher education and asked to join the control group and complete the survey at two measurement points (pre- and post-intervention).
Module 2: Situations total time 135 minutes	Students are able distinguish between intra- and interpersonal emotion regulation strategies and apply them appropriately.	5. Interpersonal ER 6. Challenging situation in teaching Duration: Approximately 30 min	(a) Balint group format: Describing personal emotions in challenging classroom situations and efforts to cope with them. (b) Transfer ERS: What ERS works in which situation best for me? Discuss with others. Duration: Approximately 105 min	
Module 3: Individual total time 135 minutes	Students reflect on their own behavioral patterns in dealing with emotions and expand their scope for action	2.1. ERS: Cognitive change (2.1.4) and response modulation (2.1.5); 7. Psychological flexibility (ACT) Duration: Approximately 30 min	(a) Relaxation techniques (2.1.5): breathing (4 in 7 out); breathing square; (b) ABC (2.1.4) and stress thoughts (c) acceptance mindset activities (7.) (d) Transfer ERS: Write down individually what works best for me. Duration: Approximately 105 min	

**Figure 2:**
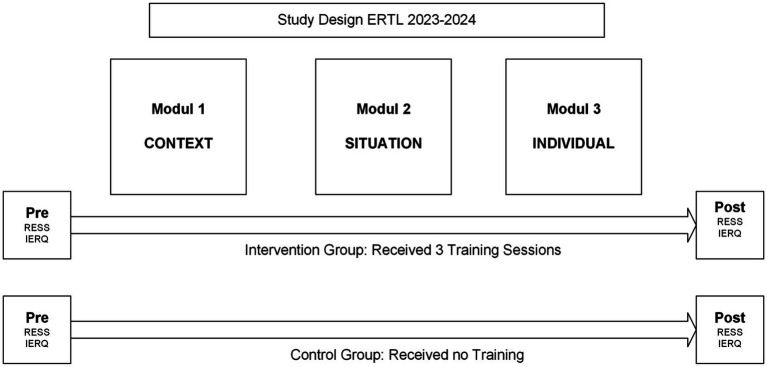
Study design.

Quantitative data were gathered using a quasi-experimental design. Participants in both the intervention and control groups completed intrapersonal ER (Regulation of Emotion System Survey; RESS) and interpersonal ER (Interpersonal Emotion Regulation Questionnaire; IERQ) measures online at two separate points (pre- and post-intervention) (see Figure 2).

#### 5.2.4 Data analysis and statistical procedures

To revise the factorial structure of the scales, confirmatory factor analyses (CFA) were conducted using the R package (R Core Team, 2017) lavaan (Rosseel, 2012). The estimation of the model parameters was performed using the diagonal weighted least squares (DWLS) estimator. To determine the goodness of fit of the measurement model, the χ^2^/df ratio, root mean square error of approximation (RMSEA), standardized root mean square residual (SRMR), Tucker-Lewis Index (TLI), and Comparative Fit Index (CFI) were used. The following cut-off values for a good model fit were used: SRMR ≤ 0.08 (acceptable fit: ≤0.1), RMSEA ≤ 0.06 (acceptable fit: ≤0.08), CFI and TLI ≥ 0.95 (acceptable fit: ≥0.9) (Hu and Bentler, 1999). The reliabilities of the individual scales were calculated using the R package semTools (Jorgensen et al., 2022). McDonald’s Omega (*ω*) was used as a measure for reliability. To examine potential baseline differences for all emotion regulation scales between the intervention and control groups, independent-sample *t*-tests or Mann–Whitney U-Tests were performed. A series of 2×2 repeated-measure ANOVAs (within-subject-factor: pre- and post ER measures; between-subject-factor: intervention group vs. control group) and simple effect analysis were used to evaluate the effect of the intervention in JASP version 0.95.4 (JASP-Team, 2025). As an effect size ω^2^ was calculated (0.01 = small effect, 0.06 = medium, 0.14 = large effect). In the case of violations of equality of variances, robust mixed ANOVAs based on trimmed means using the R package WRS2 (Mair and Wilcox, 2020) were performed. In the case of significant interaction effects, Yuen’s robust *t*-test (Yuen, 1974) from the WRS 2 package. Descriptive statistics were reported using 20% trimmed means and winsorized standard deviations (Wilcox, 2012). Robust effect sizes (for within-subject factor: robust AKP type effect size using the wmcpAKP option in R: 0.1 = small effect, 0.3 = medium, 0.5 = large effect, and robust Cohen’s d using the akp.effect option for between-subject factor and interaction: 0.2 = small effect, 0.5 = medium, 0.8 = large effect) were calculated (Algina et al., 2005; Cohen, 1988; Mair and Wilcox, 2020).

## 6 Results

### 6.1 Preliminary analysis

During the CFA with the items of the RESS, one item had to be removed due to low standardized factor loadings (≤ 0.5). The revised model after removing one item due to low standardized factor loading shows a good to acceptable model fit (χ^2^(569) = 947.915; *p* = 0.000; χ^2^/df = 1.67; RMSEA = 0.072; SRMR = 0.095; CFI = 0.963; TLI = 0.959). The model with the items of the IERQ shows a good model fit (χ^2^(154) = 221.765; *p* = 0.000; χ^2^/df = 1.44; RMSEA = 0.059; SRMR = 0.073; CFI = 0.971; TLI = 0.964). All scales of the RESS (rumination: ω = 0.85; distraction: ω = 0.91, reappraisal: ω = 0.91, relaxation: ω = 0.90, suppression: ω = 0.94, and engagement: ω = 0.88) and the IERQ (enhancing positive affect: ω = 0.74, perspective taking: ω = 0.83, soothing: ω = 0.88, and social modeling: ω = 0.91) show good reliability values.

### 6.2 Baseline differences

To examine potential baseline differences for all emotion regulation scales between intervention and control group, independent-sample *t*-tests and, in the case of violations of assumptions, the Mann–Whitney U-tests were performed. There were no significant baseline differences between the groups across the emotion regulation strategies (see Table 2).

**Table 2:** Descriptive statistics (pre) and baseline differences for the inter- and intrapersonal emotion regulation strategies of the intervention- and control group.

ER strategies	Intervention group (*n* = 86)		Control group (*n* = 42)		Baseline differences IG vs. CG
	Pre		Pre		
	*M*	SD	*M*	SD	Test statistics (df)
Intrapersonal ER strategies (RESS)					
Distraction^a^	2.97	0.93	3.15	0.66	*U*(126) = 2082.5
Rumination	3.64	0.8	3.8	0.64	*t*(126) = 1.302
Reappraisal	3.27	0.84	3.43	0.71	*t*(126) = 1.251
Arousal control	2.68	1	2.84	0.95	*t*(126) = 1.038
Suppression	2.87	0.83	2.99	0.79	*t*(126) = 0.735
Engagement	3.04	0.73	3.02	0.76	*t*(126) = −0.34
Interpersonal ER strategies (IERQ)					
Enhancing positive affect	4.25	0.62	4.25	0.63	*t*(126) = −0.015
Perspective taking	2.64	0.86	2.75	1.02	*t*(126) = 0.938
Soothing	3.1	0.9	3.07	0.95	*t*(126) = −0.12
Social modeling	3.91	0.68	3.68	0.88	*t*(126) = −1.02

IG, intervention group; CG, control group; Higher scores in the ER strategies variables indicate that the ER strategy is used more often (5-point Likert scale); ^*^ *p* < 0.05; ^**^ *p* < 0.01; ^***^ *p* < 0.001.

### 6.3 Main analysis: effectiveness of the emotion regulation intervention on intra- and interindividual emotion regulation strategies

To evaluate the effect of the intervention, a series of 2×2 repeated measures ANOVAs (within-subject-factor: pre- and post- ER measures; between-subject-factor: intervention group vs. control group) were performed (see Table 3).

**Table 3:** Pre- and post- descriptive statistics and results of the two-way mixed ANOVAs for the inter- and intrapersonal emotion regulation strategies of the intervention- and control group.

ER strategies	Intervention group (*n* = 86)				Control group (*n* = 42)				2-way mixed ANOVA					
	Pre		Post		Pre		Post		Main effect time		Main effect group		Interaction time x group	
	*M*	SD	*M*	SD	*M*	SD	*M*	SD	*F*(df)*/Q*(df)	*ES*	*F*(df)	*ES*	*F*(df)	*ES*
Intrapersonal ER strategies (RESS)														
Distraction^a^	2.97	0.93	3.05	0.78	3.15	0.66	3.42	0.79	*Q*(1, 60.98) = 3.991	–	*Q*(1, 69.76) = 5.542^*^	0.52^c^	*Q*(1, 69.76) = 0.651	–
Rumination	3.64	0.8	3.08	0.82	3.8	0.64	3.67	0.77	*F*(1, 126) = 16.687^***^	0.039^b^	*F*(1, 126) = 10.39^**^	0.036^b^	*F*(1, 126) = 5.818^*^	0.012^b^
Reappraisal	3.27	0.84	3.8	0.76	3.43	0.71	3.56	0.71	*F*(1, 126) = 19.316^***^	0.037^b^	*F*(1, 126) = 0.003	–	*F*(1, 126) = 6.698^*^	0.012^b^
Arousal control^a^	2.68	1	3.55	0.82	2.84	0.95	2.92	1.16	*Q*(1, 68.8) = 9.103^**^	0.61^c^	*Q*(1, 70.6) = 0.758	–	*Q*(1, 70.6) = 4.874^*^	0.74^c^
Suppression	2.87	0.83	2.63	0.78	2.99	0.79	2.95	0.75	*F*(1, 126) = 3.338	–	*F*(1, 126) = 2.803	–	*F*(1, 126) = 2.108	–
Engagement	3.04	0.73	3.2	0.65	3.02	0.76	2.99	0.82	*F*(1, 126) = 1.066	–	*F*(1, 126) = 1.13	–	*F*(1, 126) = 1.41	–
Interpersonal ER strategies (IERQ)														
Enhancing positive affect	4.25	0.62	4.27	0.54	4.25	0.63	4.09	0.68	*F*(1, 126) = 1.325	–	*F*(1, 126) = 0.628	–	*F*(1, 126) = 1.959	–
Perspective taking	2.64	0.86	2.61	0.79	2.75	1.02	2.6	0.92	*F*(1, 126) = 2.26	–	*F*(1, 126) = 0.296	–	*F*(1, 126) = 1.426	–
Soothing	3.1	0.9	3.18	0.86	3.07	0.95	2.98	0.92	*F*(1, 126) = 0.023	–	*F*(1, 126) = 0.43	–	*F*(1, 126) = 1.763	–
Social modeling	3.91	0.68	3.92	0.76	3.68	0.88	3.57	0.86	*F*(1, 126) = 0.772	–	*F*(1, 126) = 2.681	–	*F*(1, 126) = 1.491	–

Higher scores in the ER strategies indicate that the ER strategy is used more often (5-point Likert scale); ^*^ *p* < 0.05; ^**^ *p* < 0.01; ^***^ *p* < 0.001.

a Robust mixed ANOVAs based on trimmed means, trimmed means and winsorized standard deviations are reported in the results section; ES = effect size.

b ω^2^.

c Robust effect size for robust mixed ANOVAs based on trimmed means.

**Figure 3:**
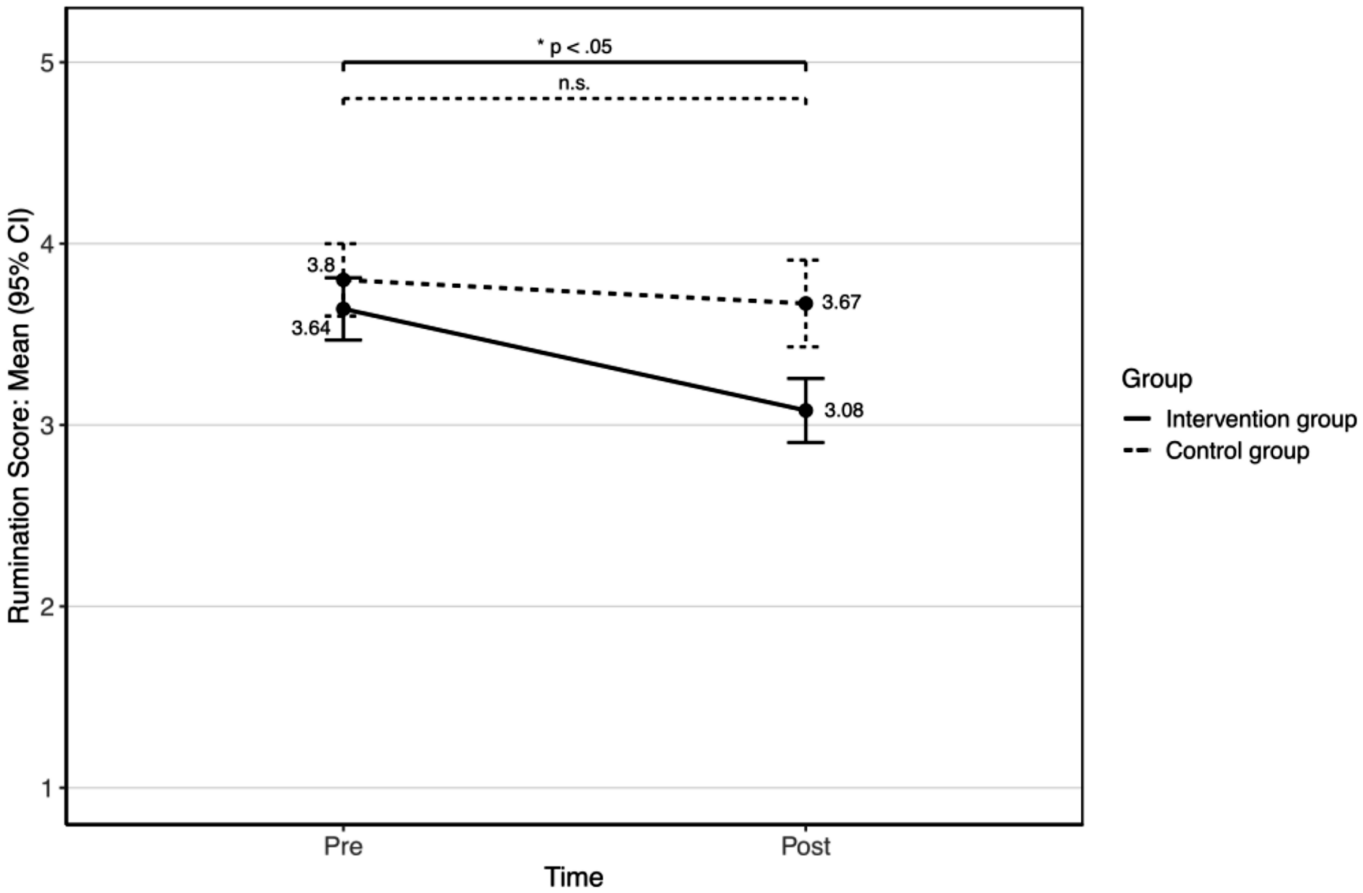
Means with 95% CI intervals of the rumination scores for the intervention group and the control group over time.

For rumination, there was a significant main effect of time (*F*(1, 126) = 16.687, *p* < 0.001, *ω^2^* = 0.039), which indicates an overall decrease in rumination scores from pre- (*M* = 3.69, SD = 0.75) to post- measure (*M* = 3.27, SD = 0.85). The main effect of group was also significant (*F*(1, 126) = 10.39, *p* = 0.002, ω^2^ = 0.036). The overall rumination score was lower in the intervention group (*M* = 3.36, SD = 0.86) than in the control group (*M* = 3.73, SD = 0.71). The interaction time x group was also significant (*F*(1, 126) = 5.818, *p* = 0.017, ω^2^ = 0.012). Simple effect analysis (see Figure 3) shows that the rumination score significantly decreases in the intervention group (*M_pre_* = 3.64, SD*_pre_* = 0.8, *M_post_* = 3.08, SD*_post_* = 0.82, *p* < 0.001), whereas no significant changes occurred in the control group (*M_pre_* = 3.8, SD*_pre_* = 0.64, *M_post_* = 3.67, SD*_post_* = 0.77, *p* = 0.249).

**Figure 4:**
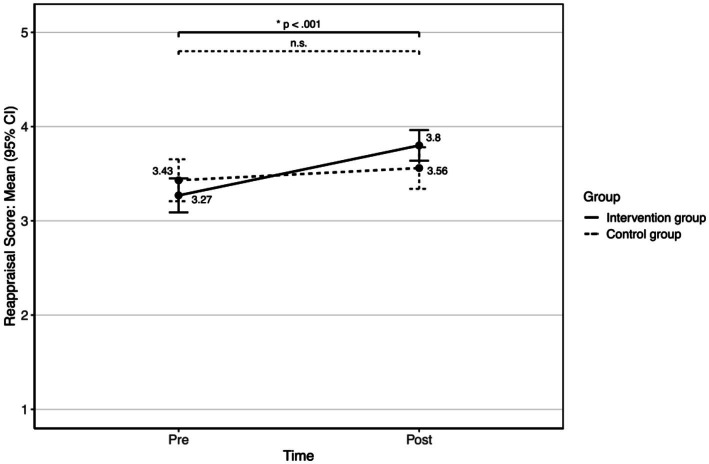
Means with 95% CI intervals of the reappraisal scores for the intervention group and the control group over time.

For reappraisal, there was also a significant main effect of time (*F*(1, 126) = 19.316, *p* < 0.001, *ω^2^* = 0.037), which indicates an overall decrease in reappraisal scores from pre- (*M* = 3.32, SD = 0.8) to post-measure (*M* = 3.72, SD = 0.75). The main effect of the group was not significant (*F*(1, 126) = 0.003, *p* = 0.956). The interaction time x group was significant (*F*(1, 126) = 6.698, *p* = 0.011, ω^2^ = 0.012). Simple effect analysis (see Figure 4) shows that the reappraisal score significantly increases in the intervention group (*M_pre_* = 3.27, SD*_pre_* = 0.84, *M_post_* = 3.8, SD*_post_* = 0.76, *p* < 0.001), whereas no significant change was found in the control group (*M_pre_* = 3.43, SD*_pre_* = 0.71, *M_post_* = 3.56, SD*_post_* = 0.71, *p* = 0.194).

No significant main or interaction effects were found for the engagement and suppression scores (see Table 2).

**Figure 5:**
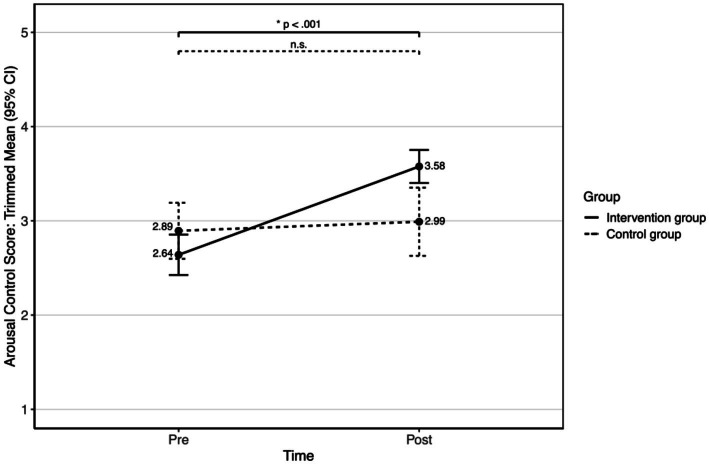
Trimmed means (20%) with 95% CI intervals of the arousal control scores for the intervention group and the control group over time.

Due to violation of homogeneity of variances, robust mixed ANOVA (20% trimmed means) were calculated for the ER strategies, arousal control, and distraction (see Table 2). For arousal control, the ANOVA shows a significant main effect of time (*Q*(1, 68.8) = 9.103, *p* = 0.004, robust effect size = 0.61), indicating an overall increase in arousal control scores over time (pre: trimmed mean = 2.73; SD = 0.76; post: trimmed mean = 3.46, SD = 0.59). The main effect of group was not significant (*Q*(1, 70.6) = 0.758, *p* = 0.387). The time x group interaction was significant (*Q*(1, 70.6) = 4.874, *p* = 0.03, robust effect size = 0.74). Alpha adjusted (Holm method) robust paired-samples *t*-test based on trimmed means (see Figure 5) shows that the arousal control scores significantly increased in the intervention group (*t*(51) = 6.953, *p* < 0.001; pre: trimmed mean = 2.64, SD = 1; post: trimmed mean = 3.58, SD = 0.82). There was no significant change in the control group (*t*(25) = 0.653, *p* = 0.52; pre: trimmed mean = 2.89, SD = 0.95; post: trimmed mean = 2.99, SD = 1.16).

For distraction, the robust mixed ANOVA shows a significant main effect of the group (*Q*(1, 69.8) = 5.542, *p* = 0.021, robust effect size = 0.52). The overall distraction score was lower in the intervention group (trimmed mean = 3, SD = 0.58) than in the control group (trimmed mean = 3.33, SD = 0.51). There was no significant main effect of time (*Q*(1, 60.9) = 3.991, *p* = 0.0502) and no significant interaction effect (*Q*(1, 69.8) = 0.651, *p* = 0.422).

The analyses of the interpersonal emotion regulation strategies (see Table 3) show that there were no significant main and interaction effects in the interpersonal emotion regulation strategies (social modeling, enhancing positive affect, perspective taking, and soothing). The intervention has no effect on the use of interpersonal emotion regulation strategies.

In summary, the results show that the intervention led to a significant reduction in the use of rumination and a significant increase in the use of reappraisal and arousal control in the intervention group with no significant changes in the control group (see Figures 3–5; Table 3). The corresponding effect sizes indicate medium effects of the intervention on the use of these emotion regulation strategies.

## 7 Discussion

The objective of this intervention study was to examine the effects of a comprehensively designed training program, ERTL, for regulating negative emotions that integrates the professional requirements and social context of the teaching profession. The findings indicated a significant impact of the intervention on reported cognitive strategies, specifically rumination and reappraisal, as well as on arousal control scores.

This intervention significantly reduced participants’ rumination. Failure to detach from negative work-related cognitions (Sonnentag, 2018) results in rumination, which is closely associated with negative emotions and subsequent emotional exhaustion (Geisler et al., 2019). Pre-service teachers often face situations that trigger negative emotions during their teaching practicum, which they tend to suppress and dwell on. This intervention significantly reduced participants’ rumination, demonstrating several benefits for pre-service teachers. They learn strategies to handle negative emotions, which help both their future careers and their academic pursuits. One explanation lies in the impact of the acceptance methods and strategies employed in ERTL. These findings are consistent with a study linking acceptance to reduced rumination (Erduran Tekin, 2022).

The antecedent-focused ERS of reappraisal offers numerous advantages for managing emotions. Participants in the training group exhibited a significantly increased utilization of ERS reappraisal. These results align with those of a comparable training program aimed at fostering socio-emotional competence in pre-service teachers (Carstensen et al., 2019), which likewise demonstrated effectiveness in cognitive reappraisal. Prior research has demonstrated that this strategy is effective in low-to-moderate stress contexts, such as instructional challenges. In contrast, in high-stress scenarios, alternative strategies such as distraction or suppression are employed (McRae and Gross, 2020).

The final observed cognitive strategy was distraction, which showed no significant interaction effect. This may be due to distraction being a typical coping strategy for novice teachers to deal with stress, and might also be because the ERS distraction was not explicitly trained in the ERTL modules with the students. Additionally, much about the ERS distraction and its effects in teaching remains unclear (Volkaert et al., 2020; Zhang et al., 2022).

There has been a relatively limited focus on the physiological regulation of arousal as a mechanism for ERS (De France and Hollenstein, 2017). This issue is particularly pertinent for novice teachers, who may find student misbehavior a high-stress situation, eliciting negative emotions such as anger. In such challenging contexts, strategies for arousal control can serve as alternatives to suppression and are more accessible than cognitive strategies in high-stress situations (Langeslag and Surti, 2017; Shafir et al., 2016). Interventions, including breathing and relaxation techniques, assist pre-service teachers in managing their negative emotions. Findings show that physical relaxation techniques, which have already been successfully used for an extended period in sports psychology (Gould and Udry, 1994; Tossici et al., 2024) and clinical settings (Peciuliene et al., 2015), expand the repertoire of ERS available to teachers. In addition, concepts from embodiment research (Robinson and Thomas, 2021) should be incorporated into the development of arousal control ERS.

The behavioral strategies investigated, suppression and engagement, did not significantly change over time in the two groups. The ERS engagement (i.e., situation modification) involves problem-solving in a negative, emotion-triggering situation and thus its modification (Jiang et al., 2016). In teaching contexts, engagement relates to classroom management techniques (Sutton et al., 2009) that may be discussed in other curriculum courses and were therefore not new to the students. It is plausible that participants in the intervention group realized that high-stress classroom situations, where no modification is possible, sometimes only allow acceptance and physiological relaxation as a last resort.

Regarding interpersonal ER, the IERQ scales (Hofmann et al., 2016) show no significant differences between the training and control groups. Participants in both groups frequently use the ERS, enhancing positive affect and social modeling at both the first and second measurement points, indicating that students typically cope with negative emotions by engaging in social events. Participants in both groups reported that the ERS perspective taking and soothing are used least often. Interestingly, a study (Han and Geng, 2025) found that soothing is negatively associated with teachers’ well-being, whereas perspective taking and social modeling demonstrate a strong positive relationship with psychological well-being.

On the one hand, these findings illustrate that research on interpersonal ER is considerably newer (Yin et al., 2025) and that the phenomenon and its distinct ERS are less well understood, so its measurement tools are therefore less refined (Williams et al., 2018). On the other hand, the results of the present study underscore the need to further adjust the ERTL training program to provide students with more time in settings that enable the exchange of perspectives and an orientation toward role models. The null effects indicate that the ERTL program is weighted toward intrapersonal ERS, with less time allocated to practicing interpersonal ERS. Mere exposure to the ERTL intervention in a group setting did not significantly change participants’ interpersonal ERS survey scores, therefore group therapy techniques need to be considered to enhance outcomes (Messina et al., 2021). Further adjustments to the training program would be useful to underscore the importance of interpersonal ER in managing emotional teaching situations.

## 8 Conclusion and implications

The present intervention study yields promising outcomes that help pre-service teachers manage their emotions adaptively. Large-scale interventions, including CARE (Cultivating Awareness and Resilience in Education) (Jennings et al., 2017; Jennings et al., 2019) and Stress and Release (Schussler et al., 2018), as well as emotional intelligence training (Gilar-Corbi et al., 2018; Özdemir Cihan and Dilekmen, 2024), report positive effects on teachers’ adaptive ER. This underscores the interrelation and substantive overlap among ER, stress, mindfulness, and emotional intelligence—considerations that should be incorporated into program design—and highlights the practical significance of a holistic approach.

The proposed CSI Model (see Figure 1) offers an encouraging framework. Teachers must develop a robust, broad set of ERS by considering contextual and situational factors and analyzing the elements within their control and preferences. Teachers’ ability to regulate emotions depends heavily on their personal experiences and is thus closely intertwined with their individual socialization processes (Takada and Dan-Glauser, 2024).

This study shows that it is imperative for pre-service teachers to contextualize subject content to specific classroom situations. Anticipated challenging scenarios should be addressed through antecedent-focused ERS (Gross, 2013). Initially, classroom situations must be selected to align with the specific class and individual students, taking into account their potential and challenges. Contextual factors such as school culture, display rules, available equipment, and class timing (e.g., schedule or late sessions) are crucial for selecting situations that enhance student learning and mitigate negative emotions in teachers. For instance, if noisy classes provoke teachers’ anger at the end of the day, this issue can be addressed by transitioning from frontal instruction to project-based learning. Consequently, lesson preparation should integrate subject content with classroom management strategies and ERS in mind. A comprehensive range of instructional and classroom management strategies helps educators in addressing negative emotions when problem-solving and situation modification are feasible. In tense classroom scenarios, expressing one’s emotions constructively while teaching can not only help regulate teachers’ emotions but also cultivate empathy among students. Moreover, pre-service teachers must learn to regulate their emotions by intentionally redirecting their attention. Instructional challenges and student misbehavior necessitate adaptable focus and diverse approaches. In addition, behavioral issues can be addressed by experienced educators by refraining from reacting prematurely and from engaging with disruptive student action (Imhof and Schlag, 2018). Our intervention program ERTL shows that in circumstances where the above strategies are not feasible, cognitive changes, such as acceptance, can enhance resilience and emotional well-being of pre-service teachers. Each individual teacher must develop a robust set of cognitive change strategies by considering contextual and situational factors and analyzing the elements within their control; physiological relaxation techniques can help teachers manage intense negative emotions during high-stress situations by providing this sense of control.

Accordingly, the teacher, as an individual and their body, perceptions, and interpretations, in the authentic implementation of ERS in the professional context. The CSI model’s differentiation of perspectives makes it possible to reflect on one’s own preferences and the subjective effectiveness of ERS, while distinguishing the roles ultimately played by context, situation, and personal characteristics. This conceptual separation of the model allows ERS to be situated accordingly and its fit with context, situation, and personality dispositions to be examined. Findings show that the intrapersonal ERS reappraisal is less effective when individuals have a high level of the trait need satisfaction (Brockman et al., 2023). This example illustrates the connection between specific strategies and personality traits, as well as the difficulty of “one-size-fits-all” solutions that fail to account for individual prerequisites and needs.

In summary, the proposed CSI Model (see Figure 1) provides a framework for addressing negative emotions in teaching settings. When teachers encounter negative emotions, they should initially identify and label the emotion, subsequently ascertain the context of its occurrence, and finally recognize the specific situation that precipitates it. These analytical steps can assist educators in identifying the most effective individual ER skills and preferences. This process enables them to develop a broad repertoire of ER strategies and to foster confidence in their ability to manage negative emotions constructively.

In particular, this intervention study found no effects for interpersonal ERS, which may reflect the construct’s greater complexity and therefore its greater difficulty to teach. Interpersonal ERS can foster pre-service teachers’ emotional well-being by providing a space for exchanging experiences, including stressful and negative ones, with other teachers, particularly mentors (Zhang et al., 2025). On the one hand, novice teachers should seek out colleagues to discuss negative emotions in teaching and strategies for handling these situations. On the other hand, school leaders need to implement professional learning communities (Gajda and Koliba, 2008) that foster exchanges to deal with challenging classroom situations. Providing a healthy school environment that brings together teachers, students, parents, and school leaders can be achieved through positive emotion-evoking experiences such as social events. This approach can be used to enhance positive affect and mitigate negative emotion.

## 9 Limitations and future research

The institutional constraints precluded the implementation of the initially planned waitlist control group design, as students were enrolled in this course as part of their standard curriculum, thereby preventing randomization. Future research should evaluate the proposed intervention model in this context and ensure randomization of groups before the intervention commences. Furthermore, in accordance with curricular guidelines, the present intervention comprises three modules over three weeks. This relatively short period without follow-up measurements limits long-term sustainability. A design-based research (DBR) approach (Schmiedebach and Wenger, 2021) will further develop the prototype of the ERTL intervention during this initial evaluation phase, so that it can be implemented as a broader intervention with five modules to work through the CSI model’s content with students and three measurement points in the future.

Future research should continue exploring tailored interventions that address teachers’ specific needs while accounting for individual differences in emotion regulation strategies (Yin et al., 2016; Braun et al., 2020; Byun and Jeon, 2023). These differences can include the systematic impact of demographic factors such as gender, which have already been shown to play a role in emotion regulation (Ford and Mauss, 2015). Results from such future research could also be integrated into the CSI model and the ERTL intervention to provide a more robust foundation and a nuanced match.

## Data Availability

The raw data supporting the conclusions of this article will be made available by the authors, without undue reservation.
